# A New Conformal Cooling System for Plastic Collimators Based on the Use of Complex Geometries and Optimization of Temperature Profiles

**DOI:** 10.3390/polym13162744

**Published:** 2021-08-16

**Authors:** Jorge Manuel Mercado-Colmenero, Abelardo Torres-Alba, Javier Catalan-Requena, Cristina Martin-Doñate

**Affiliations:** Department of Engineering Graphics Design and Projects, University of Jaen, 23071 Jaen, Spain; jmercado@ujaen.es (J.M.M.-C.); ata00001@red.ujaen.es (A.T.-A.); jcr00020@red.ujaen.es (J.C.-R.)

**Keywords:** conformal cooling, injection molding, numerical simulation, injection mold design, sustainability, industrial design

## Abstract

The paper presents a new design of conformal cooling channels, for application in collimator-type optical plastic parts. The conformal channels that are presented exceed the thermal and dynamic performance of traditional and standard conformal channels, since they implement new sections of complex topology, capable of meeting the high geometric and functional specifications of the optical part, as well as the technological requirements of the additive manufacturing of the mold cavities. In order to evaluate the improvement and efficiency of the thermal performance of the solution presented, a transient numerical analysis of the cooling phase has been carried out, comparing the traditional cooling with the new geometry that is proposed. The evolution of the temperature profile versus the thickness of the part in the collimating core with greater thickness and temperature, has been evaluated in a transient mode. The analysis of the thermal profiles, the calculation of the integral mean ejection temperature at each time of the transient analysis, and the use of the Fourier formula, show great improvement in the cycle time in comparison with the traditional cooling. The application of the new conformal design reduces the manufacturing cycle time of the collimator part by 10 s, with this value being 13% of the total manufacturing cycle of the plastic part. As a further improvement, the use of the new cooling system reduces the amount of thickness in the collimator core, which is above the ejection temperature of the plastic material. The improvement in the thermal performance of the design of the parametric cooling channels that are presented not only has a significant reduction in the cycle time, but also improves the uniformity in the temperature map of the collimating part surface, the displacement field, and the stresses that are associated with the temperature gradient on the surface of the optical part.

## 1. Introduction

Lighting fixtures that are equipped with LED lamps provide high-quality and efficient white light, reducing the device’s energy consumption by up to five times less than the consumption of conventional fluorescent lamps. This fact has led to an exponential increase in the use of LED lighting in the recent years, in areas as diverse as the instrumentation for light measurement, spectrometers, and especially in the automotive lighting sector [1]. LED technology is linked to the use of optical elements that are capable of redirecting, concentrating, and taking advantage of the LED light, avoiding glare, and improving the performance of the luminaire [2,3]. The design of the optical elements for LED light prevents the light from being flat, diffusing, and being of little use [4]. The optical elements are classified into the following three large groups: primary, secondary, or tertiary; this depends on their positioning with respect to the LED light. Collimating lenses are found in the group of secondary optics, and are in charge of shaping the light, expanding the light beam, or concentrating it on a specific point. The low operating temperatures in LED head lamps allow the use of transparent polymers to replace materials such as glass in the manufacturing of collimating lenses [5]. This fact encourages more and more automobile manufacturers to use injection molded thermoplastics to produce collimating lenses with complex geometries for the design of new automotive headlights.

Plastic collimating lenses require precision manufacturing with high dimensional tolerances, avoiding any small warping in the geometry of the piece [6,7,8,9]. The collimating lens topology is characterized by its variable geometry, as well as its high thickness ratios, understanding this ratio as the relationship between the maximum and the minimum thickness of the lens [10]. These technical specifications are a challenge for injection mold designers, since as the wall thickness increases, so does the shrinkage potential of the plastic part. For this reason, reliably molded collimating lenses within the range of dimensional accuracy, required in the automotive industry, are very difficult to manufacture. Additionally, the manufacturing of optical lenses with large variations in thickness causes accumulations of heat in specific areas of the piece, due to a slower cooling process [11]. This fact, as a consequence, increases the total cooling time, as well as a lack of uniformity in the cooling along the geometry of the piece, which can cause a loss of the optical properties of the lens. Additionally, and in line with energy savings, the accumulation of heat in the plastic lens will greatly influence the productivity of the industrial process that is so important in the automotive field.

The optimization of the injection molding process, either by improving the product quality or reducing the injection cycle time, depends largely on the design of the mold and the geometry of the plastic part to be manufactured [12]. The cycle time in the production of a mold is a measure of its productive efficiency [13,14]. A small decrease in the industrial cycle time leads to a large decrease in the energy expenditure of the process, making it more efficient and sustainable [15]. However, decreasing the cycle time, while maintaining the quality and requirements that are specified by the client, is a highly complex process that involves the detailed study of the heat exchange process between the surface layers, internal areas of the piece, and the flow that circulates through the cooling channels.

With respect to the injection cycle, the cooling stage is the most time-consuming component of the total cycle [16]. In addition, it has a great influence on the final properties of the plastic part, so cooling must be as efficient as possible. Injection molding cooling channels are traditionally manufactured using subtractive technologies, such as CNC. The use of these technologies requires the fulfillment of a series of dimensional criteria that are necessary to guarantee the structural integrity of the mold against the high pressures and efforts to which it will be subjected during its productive life. Additionally, subtractive technologies present high levels of material waste, so unfortunately they are not in line with the current sustainability requirements [17,18,19]. On the contrary, the additive manufacturing process allows greater freedom in the manufacturing and design of conformal channels, adapting the channel topology to the requirements of the geometric surface of the plastic part [20]. In this way, it is possible to eliminate, or reduce, the existence of hotspots that are caused by differences in the thickness, accumulations of material, or deep areas that are difficult to cool.

The layout, topology, and sizing of the cooling channels have a great influence on the variables of the heat exchange process, such as pressure drop, cooling efficiency, coolant flow speed, etc. The design of conformal cooling channels in the injection mold involves determining the layout of the cooling channels as well as the geometry of the channel section [21]. Although the circular cross section is the most common in the design of conformal channels, some researchers have developed conformal channels with non-circular cross sections, such as square, rectangular, rhomboid, elliptical, raindrop, etc. [22]. The use of a square section for the cooling channels has been used by several authors, by making cooling slots in the mold [23]. The implementation of this solution is viable through traditional methods; however, it is difficult to do with additive manufacturing, since it requires supports that prevent the collapse of the material in the upper zone of the channel. In order to avoid deviations with the circular surface of the channel or even collapse in the upper zone, Kamat et al. [24] modified the circular section to a triangular self-supporting teardrop profile. The semicircular-shaped cooling channels consist of the following two parts: a first semicircular part and another straight part that is parallel to the contour of the cavity. Unfortunately, although the semicircular conformal channel allows better tracking of the surface part versus the circular conformal channel, and an improvement in heat dissipation [25,26], the sharp corner at the junction of the semicircular portion and the straight portion can cause stress concentration and crack propagation. The layout of the conformal cooling channels involves determining the sweep geometry of the channels with respect to the part to be manufactured; in this line, several authors have presented various geometric proposals for conformal cooling channels. Spiral geometry is one of the most used topologies in the design of the standard layout in conformal cooling channels [27]. Linear zigzag geometry [28,29] is used in cases where spiral circuits are difficult to implement. It should be noted that spiral geometry versus zigzag topology features sharp turns, increasing the pressure drop, slowing the flow rate, and thus weakening the cooling efficiency. The application of the spiral-shaped and zigzag conformal channels decreases as the difficulty of the geometric surface of the plastic part to be manufactured increases. In these cases, the mesh topological conformal channels [30], the lattice [31], the conformal porous channels [32], and the vascularized conformal systems [33], can be applied for complex parts. Unfortunately, the design of a lattice conformal cooling system requires detailed study regarding flow distribution and pressure drop, since the usual design rules are useful for channel geometries with uniform diameters and shapes. In line with the admissible pressure drop in the circuit, there is a minimum channel diameter below which the channel cannot be divided into sub-branches [34]. Also, from a functional point of view, in the event of an obstruction situation in the channels, by foreign bodies, it would be difficult to extract them due to the communication of the channels between them.

Although the concept of conformal cooling allows the design of channels with freeform geometry, its manufacture is limited by a series of design criteria for additive manufacturing [35,36,37]. The design of freeform channels, for application in complex geometries, requires an optimization process in which it is possible to maximize the thermal exchange by reducing the cooling time, while meeting the manufacturing criteria that prevent the collapse of the material, such as the use of internal supports.

The geometry of the collimating optical elements presents multiple geometric limitations that prevent the use of standard conformal channels. As they are optical and aesthetic parts at the same time, their dimensions are minimal, being plastic parts of very small dimensions. The optical requirements force the design of collimators with concentrating topologies of the initial LED light. These concentrators require high-thickness walls and conical shapes joined together, which limits the space for the placement of standard conformal cooling channels. The accumulation of thicknesses in localized areas of the part causes hot spots and differential shrinkage of the material, which requires long cooling times that multiply the production cost and the energy expenditure of the process.

To solve the raised problems, the paper presents a new design of conformal cooling channels for application in collimator-type plastic optical parts. The presented conformal channels exceed the thermal and dynamic performance of traditional and standard conformal cooling channels, since they implement new sections of complex topology that are capable of complying with the high geometric and functional restrictions of the optical part, as well as with the technological requirements for the additive manufacturing of mold cavities. In this way, the presented system optimizes the heat exchange process between the cooling fluid and the plastic material. The new geometry that is presented in the paper improves the state-of-the-art, since it optimizes the heat exchange between the fluid and the surface of the part in those hot spots of the collimating part that are impossible to cool with standard conformal cooling layouts.

## 2. Materials and Methods

### Analysis of the Optical Collimator Part Manufactured through Injection Molding Technology. Geometrical Design

In this section, the geometrical, functional, and manufacturing features associated with the plastic collimator under study are described. Technical details regarding the selection of the plastic material for the injection molding manufacturing process, boundary conditions, and geometrical features are also specified.

**Figure 1:**
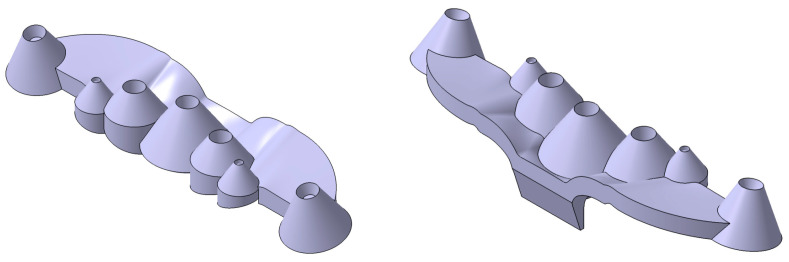
Collimating optical part.

**Figure 2:**
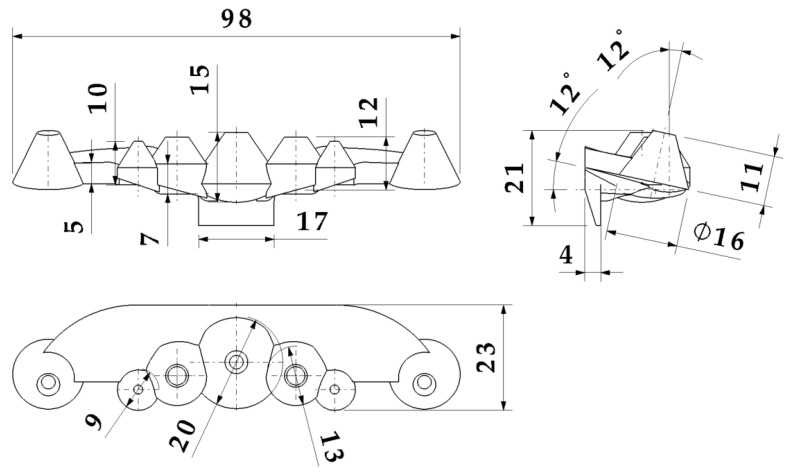
Dimensions of the collimating optical part.

The optical part under study is made up of a set of seven truncated conical collimators, with heights between 10 and 15 mm and base diameters between 9 and 20 mm. The collimators present variable lateral slopes with intersections in their base zone, which produce an overlap of the collimating surfaces. The optical part has a small topology with a 98 × 23 × 21 mm bounding box. The vertical axis of the five central collimators is perpendicular to the horizontal plane of the optical piece, while the two end collimators have an inclination angle of 12 degrees with respect to the ZX coordinate plane. The seven collimators are located on a base freeform platform in the optic piece, having a thickness of 5 mm. The gate of the optical part is located on a slanted flange centered on the lateral end of the base platform (see Figure 1). Figure 2 details the dimensions of the collimating optical piece presented in the paper.

The complex geometry of the collimating optical part presents great challenges and limitations from the point of view of its manufacture. The thickness ratio of the piece understood as the ratio between the maximum thickness of the piece and the minimum thickness is 15:4. This ratio makes it extremely difficult to design uniform cooling by increasing the cooling time. Likewise, the piece has intersection areas at the base of the collimating truncated cones, which produce significant accumulations of material. This accumulation of thicknesses can greatly influence the warping of the optics, preventing the piece from meeting the photometric requirements demanded by the client. The gate of the collimating piece is located on a lateral flange with a thickness of 4 mm. The flange is aligned with the thickest area of the piece, creating a hot spot that is very difficult to cool.

The intersection between the collimating cones together with the small dimensions of the optical piece prevents the use of traditional channels in the core of the mold as well as spiral or zigzag standard conformal channels. The base platform on which the collimators are located has a lateral inclination of 12 degrees. This fact requires the cooling in the cavity plate to be adapted to the inclination requirements of the geometry.

**Figure 3:**
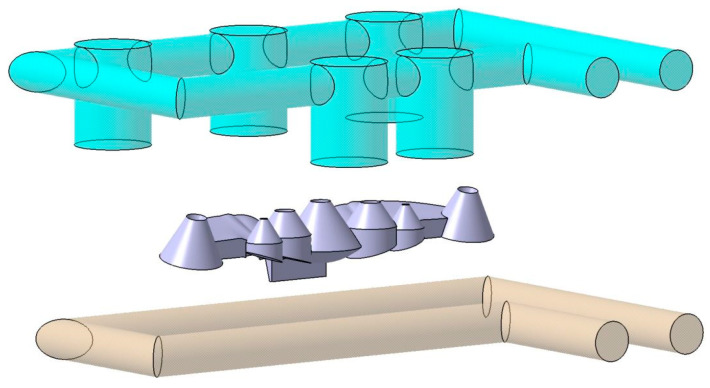
Traditional cooling solution for the collimating optical part.

The material used in the manufacturing of the collimating optical part is PC Makrolon LED 2245 [38]. Polycarbonate allows the design and manufacturing of optical parts with complex geometries for LED applications that are impossible to manufacture in glass. Figure 3 presents the traditional solution used for cooling the collimator piece. Figure 3 shows how the baffles fail to adequately cool the conical areas of the collimator since they require an uneven and not uniform location with respect to the surface of the piece. Figure 3 shows how the cavity plate is cooled by using straight channels. Compliance with traditional cooling sizing criteria forces the straight channels of the part surface to be separated by a distance large enough to prevent heat transfer between the cooling fluid and the part.

In order to solve the design problems that traditional straight channels and standard conformal channels currently present in the cooling of collimating optical parts, the paper presents a new design of conformal channels for plastic collimators based on the use of complex geometries and optimization of the temperature profiles. The complex geometries developed are capable of meeting the level of technological, functional, and geometric requirements demanded for the manufacturing of this type of optical part.

**Figure 4:**
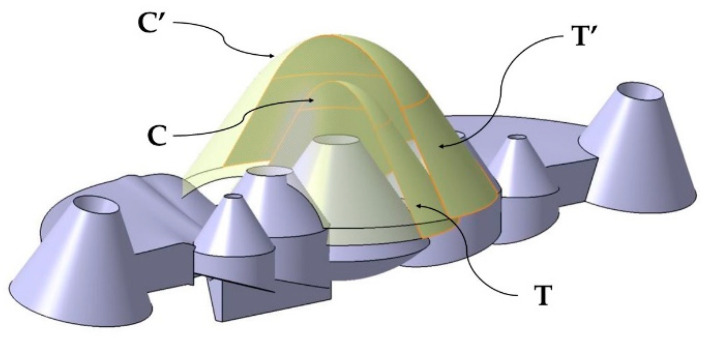
Truncated conical surfaces T, T′ and spherical domes C, C′.

**Figure 5:**
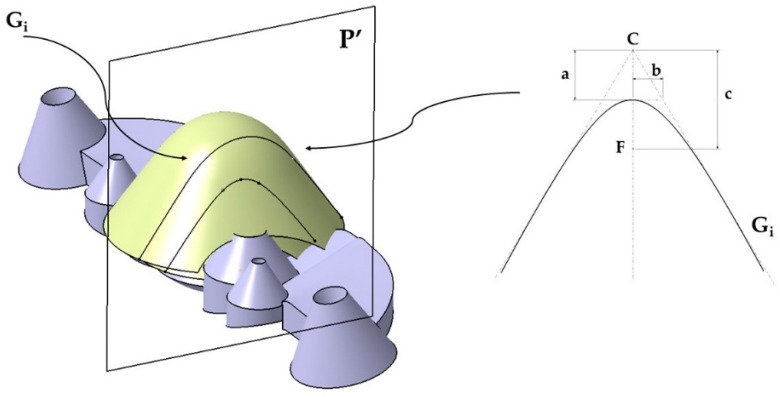
Representation of the curves G_i_ obtained from the intersection of the plane P′ with the surfaces T, T′, C, C′.

The geometry of the new conformal channels consists of two parallel complex surfaces B, B′ obtained from two generating curves S_j_ ∀ i [1,2] and two guide curves G_i_ ∀ j [1,2]. To obtain the guide curves G_i_, two truncated conical surfaces T, T′ have been modeled parallel to the surface of each collimator of the optical part. The surfaces T, T′ incorporate two spherical domes C, C′ in the upper part. In Figure 4 the base surfaces T, T′ as well as the domes C, C′ are indicated. A plane P′ parallel to the coordinate plane YX, intersects the complex surfaces T, T′ and C, C′, obtaining as a result two hyperbolic guide curves G_i_. The parameters and analytical definitions of the guide curves G_i_ are indicated in Figure 5 as well as in Equations (1)–(3) where ε is the eccentricity of the guide curve G_i_, *a* the length of the major axis of the curve G_i_ and *b* the length of the minor axis of G_i_. The parameter *c* represents the distance from the center C to the focus F of G_i_. Figure 5 shows the G_i_ curves obtained from the intersection of the plane P′ with the surfaces T, T′, C, C′.

(1) $$\frac{{\left(x-x_{0}\right)}^{2}}{a^{2}}-\frac{{\left(y-y_{0}\right)}^{2}}{b^{2}}=1\;$$

(2) $$\varepsilon =\frac{c}{a}$$

(3) $$c=\sqrt{a^{2}+b^{2}}$$

**Figure 6:**
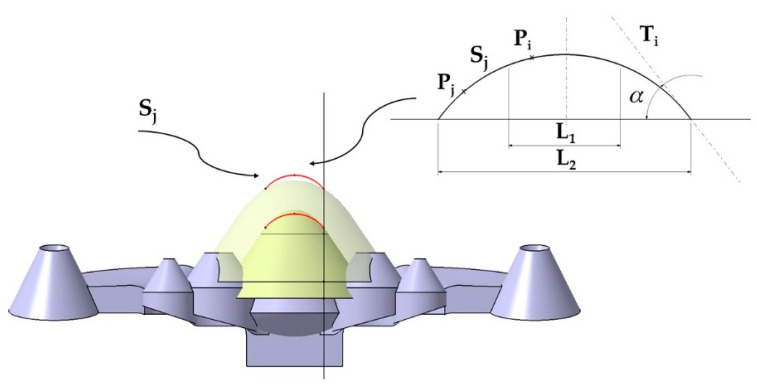
Generating curve S_j._

The generating curve S_j_ consists of two well-differentiated areas L_1_ and L_2_ (see Figure 6). In order to avoid the use of supports in the manufacturing of the conformal channels, the generating curve S_j_ consists of a central zone L_1_ formed by the locus of the points P_i_ whose tangent line T_i_ presents an inclination with the horizontal plane of less than 45°. It is crucial to make sure that the value of L_1_ does not exceed 10 mm in any case. In this way, material collapses are avoided in the fabrication of the conformal channel [19]. The second part of the curve S_j_ is formed by the set of points P_j_ belonging to the geometric locus L_2_, see Figure 6. The points P_j_ ∈ to L_2_ fulfill the condition that the inclination of the tangent line to S_j_ at each point P_j_ must exceed 45 degrees. In this way, it is possible to adapt the design of the surface of the conformal channels to the truncated conical geometry of the collimating piece without the need to use supports for its manufacture.

**Figure 7:**
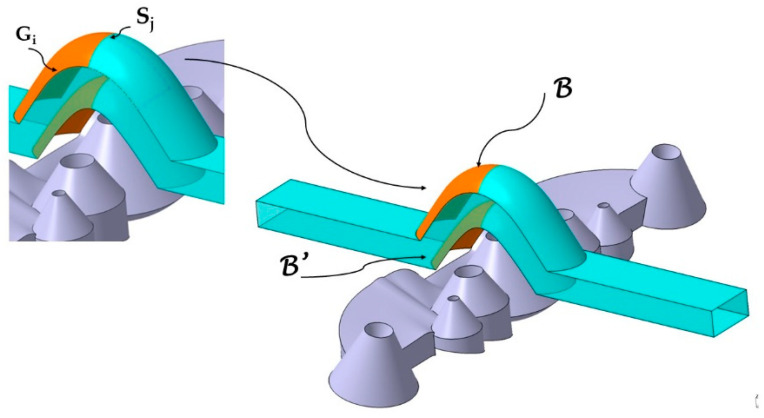
End surfaces B, B′ of the central conformal channel obtained from the sweep of the generating curves S_j_ along the guide curves G_i_.

**Figure 8:**
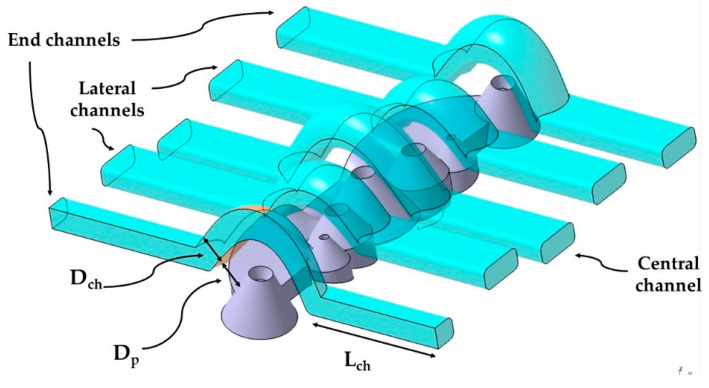
Parameters D_p_, D_ch_, y L_ch_ on the design of the new conformal channels.

The generating curves S_j_ and the guide curves G_i_ are the basis for obtaining the complex surfaces B, B′ superior and inferior to the conformal channel in each collimator. The objective is to design surfaces B, B′ that allow the obtention of conformal cooling channels with a constant section and that, at the same time, are adaptable to the geometry of each collimator. Additionally, the newly designed conformal channels meet DfAM manufacturing criteria [39]. Figure 7 shows the surfaces B, B′ of the central conformal channel obtained from the sweep of the generating curves S_j_ along the guide curves G_i_. The same geometric procedure has been followed with the rest of the collimators, obtaining two lateral channels and two end channels as indicated in Figure 8.

**Figure 9:**
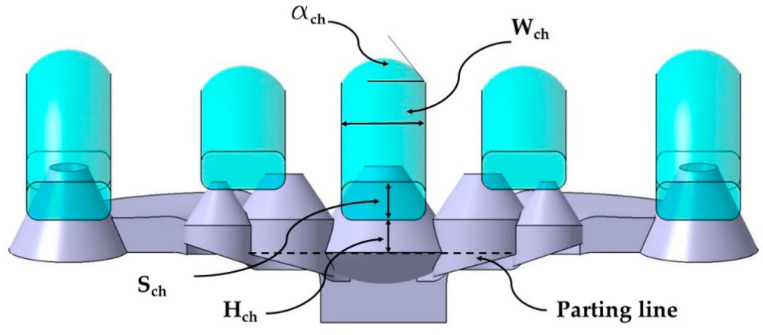
S_ch_, H_ch_, a_ch_ and W_ch_ parameters on the design of the new conformal channels.

The new design of the conformal channels has been carried out using a variable parametric model, with the aim of adapting them to the optimal measurements of each collimator. The set of parameters of the new conformal cooling design for collimators presented in the paper is indicated in Table 1. Figure 8 shows the parameters D_p_, D_ch_, y L_ch_ on one of the conformal channels. In Figure 9 the parameters S_ch_, H_ch_, a_ch_ y W_ch_ on the design of the new conformal channel are indicated.

**Figure 10:**
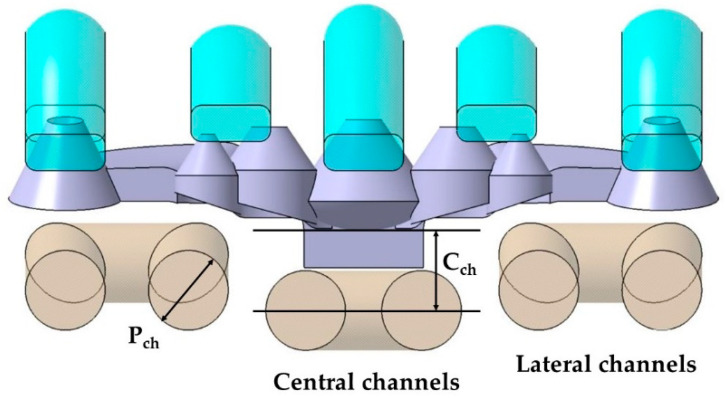
Design parameters P_ch_ y C_ch_ of the conformal refrigeration layout in the cavity plate.

**Figure 11:**
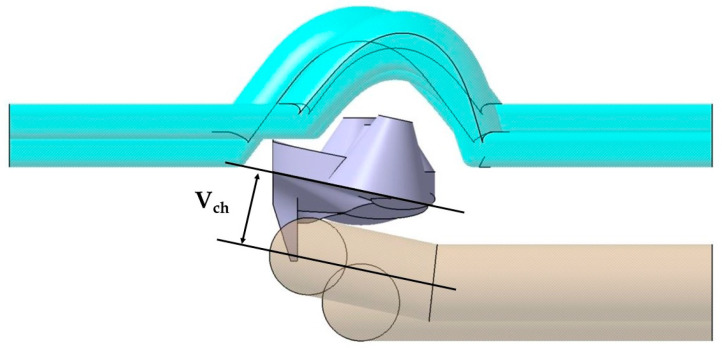
V_ch_ parameter.

**Figure 12:**
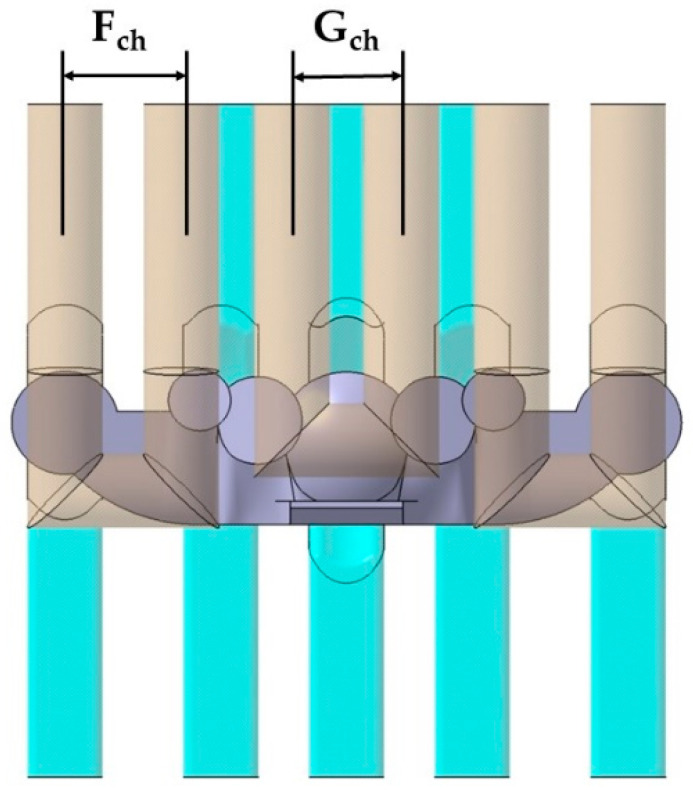
F_ch_ and G_ch_ parameters.

The base of the optical collimators responsible for controlling the LED light is characterized by a spherical dome-shaped geometry in the central collimator, combined with the use of inclined plane geometries in the lateral collimators (see Figure 10). This area requires high levels of surface quality by being molded in the cavity plate of the mold. In order to achieve a cooling that is as uniform and adapted as well as possible to the base of the collimators, a conformal cooling layout has been designed with a circular section and an inclined profile bordering the curved areas of the collimator piece. The objective is to achieve, together with the conformal cooling of the core cavity, the most possibly uniform cooling for the collimating piece that minimizes the warpings caused by a differential shrinkage in areas with a high thickness ratio as much as possible. A series of design parameters capable of obtaining the optimal configuration of the channels at the base of the collimators has been established in the same way as in the conformal layouts designed for the core cavity. The parameters for the cavity plate cooling system are shown in Table 2. Figure 10 graphically indicates the design parameters P_ch_ y C_ch_ of the conformal cooling layout in the zone of the cavity plate. Figure 11 indicates the location of the V_ch_ parameter and Figure 12 represents the F_ch_ and G_ch_ parameters.

Table 3 indicates the dimensional values of the design parameters used in the cooling of the collimating piece in the core plate of the mold. Table 4 indicates the dimensional values of the design parameters used in cooling the collimating piece in the cavity plate of the mold.

## 3. Implementation and Results

The methodology proposed in this manuscript has been developed in the CAD design software Catia (V5-6R2020 version, Dassault Systèmes, Vélizy-Villacoublay, Francia) [40] and the numerical analysis software Moldex3D (R17 version, CoreTech System Co., Ltd., Zhubei City, Taiwán) [41], with an MSI notebook with an Intel (R) Core (TM) i- 77700HQ CPU @ 2.80 GHz. As indicated in the item materials and methods, the geometry of the cooling channels of the conformal type that is presented in the paper has been parameterized and adapted to the topology of the case study and the technological requirements imposed by the 3D additive manufacturing process, selective laser sintering (SLM). Because of the geometrical freedom of metal rapid tooling methods, such as selective laser sintering (SLS) and selective laser melting (SLM), conformal cooling channels can be manufactured with flexible cross sections and topologies [39].

The applied methodology, together with the parameterization of the geometric variables of the cooling channels, is applicable to the topologies of collimating pieces. The parameterization mentioned, together with the modeling of the layout of the conformal cooling channels, has been automated by generating an application in the programming environment of the CAD software Catia V5-6R2020 [40].

### 3.1. Description of Thermal and Dynamic Modeling of Numerical Simulations

**Figure 13:**
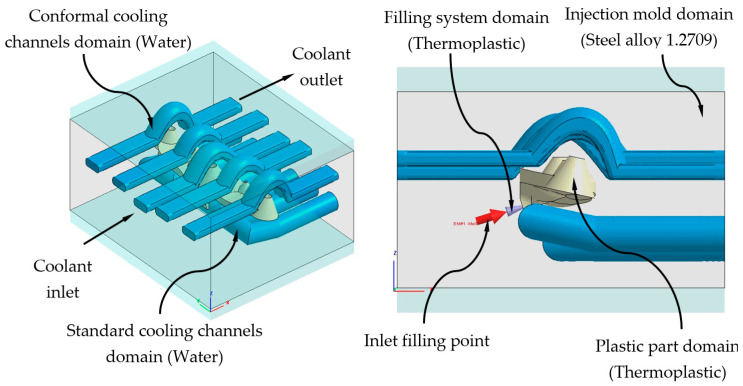
Domains and boundary conditions definition for the numerical simulations, conformal cooling solution.

**Figure 14:**
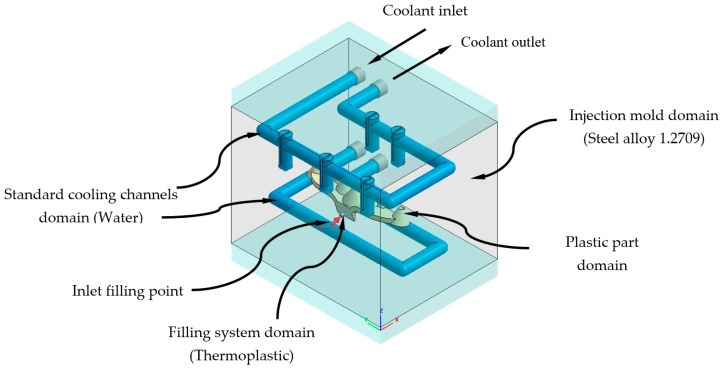
Domains and boundary conditions definition for the numerical simulations, standard cooling solution.

This section describes the configurations defined to carry out the analysis and numerical simulations, from which the thermal and rheological behavior of the geometries and designs of the cooling systems is evaluated (see Figure 8 and Figure 10). By means of this set of numerical simulations, the thermal and dynamic behavior, developed by the coolant flow, is calculated and analyzed, as well as the thermal exchange produced between them, the plastic part, and the injection mold during the cooling phase. In this way, it can be verified and validated if the thermal performance of the results obtained meets the technological requirements demanded by the manufacturing process using plastic injection molds. First, in order to carry out this set of numerical simulations, four main computational domains or 3D solids must be established (see Figure 13 and Figure 14), along with their corresponding material definition. These computational domains are defined as follows: plastic part (PC-Makrolon LED 2245), power system (PC-Makrolon LED 2245), cooling system (water), and injection mold (steel alloy 1.2709). Table 5 and Table 6 show the main physical, rheological, and thermal properties of the materials that were used for each computational domain.

Likewise, together with the definition of the main 3D solids or computational domains that define the complete geometry of the numerical analyses, a set of main hypotheses must be established, prior to carrying out the numerical simulations, as follows:

- Given that the main objective achieved with the numerical simulations is to evaluate the evolution of the temperature map of the plastic part, the evolution of the temperature map on the surface of the injection mold, the heat transfer between both domains, and the technological parameters of the cooling system as a function of the time variable, the typology of the numerical analyses carried out has been defined as “cooling transient”.
- The total time, corresponding to the cooling phase, defined for the different numerical simulations carried out, is 120 s. Throughout said cooling time, steps or time intervals are established every 10 s. In this way, for each time step, the commercial software stores the different solutions obtained. This allowed the progressive evaluation of the thermal and dynamic results that were obtained from each numerical simulation, throughout the cooling phase of the plastic part.
- The analysis of the behavior and evolution of the physical, dynamic and thermal properties of the coolant flow, along the channels of the cooling system, have been analyzed and modeled according to the “Run 3D cooling channels” configuration; this was included in the commercial software that was used to carry out the numerical analyses and simulations.
- For the configuration of the solver that was used in the numerical simulations, the maximum variation in the temperature of the surface of the mold was established as a parameter used as a convergence criterion. Thus, the convergence condition for calculating the numerical solution was defined for a temperature difference or gradient equal to 1 °C and a maximum number of cycles equal to 10.
- The turbulence model that was used for the development of the numerical analyses was established using the roughness parameter. This parameter defines the contact or the interface surface between the coolant flow and the walls of the channels of the cooling system. Likewise, according to the injection mold metal material and the manufacturing process that was defined for the manufacture the conformal cooling channels, the magnitude defined for this technological parameter is equal to 0.02 mm.

**Figure 15:**
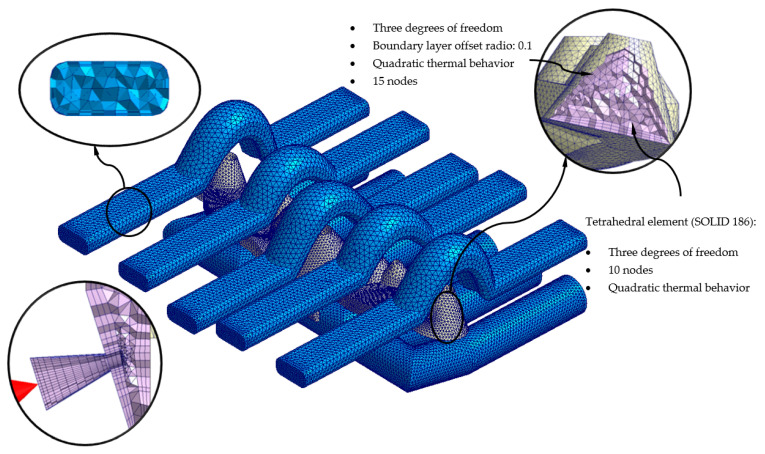
Mesh details for the conformal cooling solution.

To carry out the different numerical simulations, the main geometry of the cooling channels, plastic part, and injection mold must be previously discretized in finite volumes. The commercial software Moldex 3D R17 [40] has a Moldex Designer mesh module, in which the geometric and technological parameters of the mesh can be configured and established. Table 7 shows the magnitude of the geometric parameters defined during the meshing process, as well as its configuration. The magnitude of these parameters has been established according to the details and geometric features of the plastic part. As shown in Figure 15, the elements that were used as finite volumes, to discretize the proposed geometries, are of the second-order tetrahedron-type (SOLID 186). This means that said elements have the following 10 control nodes: 4 located at the vertex of the element and 6 located in the center of the edges that compose it. Each control node has 3 degrees of freedom, with translation on the main X, Y, and Z axes. The use of this type of element allows the resulting field of displacements and temperatures to be modeled with greater precision.

**Figure 16:**
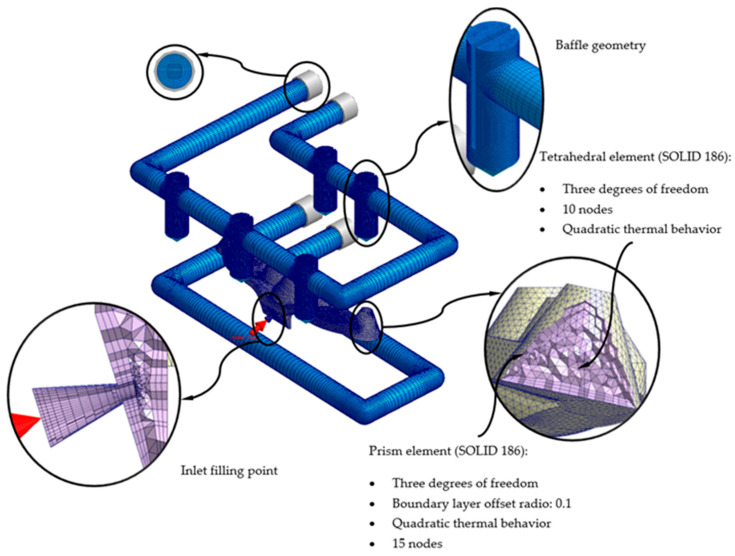
Mesh details for the traditional cooling solution.

Likewise, with the aim of improving the precision of the numerical analyses, prismatic elements of the second order (SOLID 186) of type “boundary layer mesh” (see Figure 15 and Figure 16) are defined for the interface surfaces between first the cooling channel and the injection mold, and, secondly, the plastic part and the injection mold. These have the following 15 control nodes: 6 located at the vertices of the element and 9 located in the center of the edges that compose it. Each control node has 3 degrees of freedom, with translation in the main axes X, Y, and Z. The size of these elements is established from an offset ratio, which represents a percentage of the mean size of the mesh element (see Table 7, Figure 15 and Figure 16). For the geometry of the cooling channels, five boundary-layer elements are established, to improve the modeling of the roughness between the coolant flow and the surfaces of the cooling channels (see Figure 15 and Figure 16). For the geometry of the plastic part, three boundary-layer elements are established, to improve the modeling of the thin plastic layer that solidifies when the molten plastic front comes into contact with the surface of the injection mold (see Figure 15 and Figure 16).

As shown in Figure 15 and Figure 16, a series of boundary conditions are established for each numerical simulation performed. For cooling channels, an inlet and outlet surface of the coolant flow is defined, as well as the magnitude of the technological parameters of the inlet temperature and pressure of the coolant flow (see Table 8, Figure 15 and Figure 16). The inlet temperature for the coolant flow is equal to 80 °C. It should be noted that for the dimensioning of the input pressure variable of the coolant flow to the cooling channels, a magnitude that ensures and allows the coolant flow front to develop in a turbulent regime has been established. That is, the Reynolds number of the coolant flow along the cooling channels is greater than 1.5 × 10^4^. On the other hand, for the feeding system, the input surface of the molten plastic front is established, as well as the magnitude of the technological parameters of the temperature, pressure, and flow of the molten plastic front (see Table 8, Figure 15 and Figure 16). The inlet temperature for the melt plastic flow is equal to 290 °C and the maximum injection pressure is equal to 160 MPa. Besides, the initial temperature of the computational domain corresponding to the injection mold is equal to 80 °C. It should be noted that the magnitudes selection of the initial temperatures, in each computational domain, is established by the recommendation of the thermoplastic material manufacturer.

### 3.2. Calculation of the Time to Reach the Ejection Temperature of the Plastic Part and Molten Core Analysis

The objective of this manuscript is focused on modeling, designing, and projecting a new parametric design of cooling channels, of the conformal type, which improves the thermal and dynamic performance of the current cooling system that is presented by the injection mold for the plastic part object of study. In this way, a technological parameter that determines the performance and efficiency of a cooling system of an injection mold, is the time that elapses from the beginning of the injection mold cavity filling phase until the plastic part reaches the ejection temperature. This period of time covers different phases within the manufacturing cycle of a plastic part, among which the filling, packing, and cooling phase stand out; the time associated with the cooling phase is the most important. This is due to the fact that this phase covers the highest percentage of time during the complete manufacturing cycle of the plastic part and is, therefore, the most representative. According to Menges et al. [42], to define this technological parameter, the equation that determines the one-dimensional heat flow or Fourier equation reduced to one dimension is established (see Equation (4)).

(4) $$\frac{\partial T}{\partial t}=\alpha_{s}\cdot \frac{\partial^{2}T}{\partial^{2}z}\;$$

where α_s_ [m^2^/s] represents the coefficient of the thermal diffusivity of the metal material of the injection mold. To continue with the development of the Fourier equation, reduced to one dimension (see Equation (4)), the following premises are established:

- Upon completion of the injection mold cavity filling phase, the temperature of the molten plastic front remains constant and equal to the injection temperature of the plastic part, T_melt_ [°C] (see Table 8).
- From the moment corresponding to the completion of the mold cavity filling phase, the temperature on the surface of the cavity begins a process of transient variation until reaching a stationary temperature or solution with a constant value equal to T_mold_ [°C] (see Table 8).

In this way, and from the premises previously defined, the temperature of the molten plastic front, for a point of the injection mold cavity (see Equation (5)), is defined as a particular solution, after evaluating Equation (4) as a convergence of the Fourier series development.

(5) $$T_{z=0}\left(t\right)=T_{\mathrm{coolant}}+\left(T_{\mathrm{melt}}-T_{\mathrm{coolant}}\right)\cdot \overset{\infty }{\underset{m=0}{\sum }}\frac{{\left(-1\right)}^{m}}{2\cdot m+1}\cdot e^{\frac{\pi \cdot {\left(2\cdot m+1\right)}^{2}\cdot \alpha_{s}}{T_{p}^{2}}\cdot t}$$

**Figure 17:**
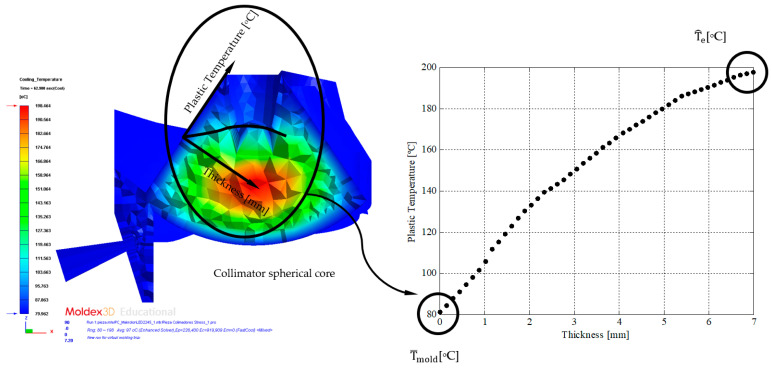
Distribution of the temperature profile of the plastic part [°C] versus the thickness [mm] in the collimator region.

Likewise, solving Equation (5) with respect to the cooling time variable, and assuming that the geometric region of the plastic part with the longest cooling time is found in the spherical core of the collimators (see Figure 17) where the largest diameter of the plastic part is located, Equation (6) is defined as the expression that determines the total cooling time of the plastic part under study.

(6) $$t_{\mathrm{cooling}}=\frac{D^{2}}{4\cdot \pi^{2}\cdot \alpha_{s}}\cdot \mathrm{Ln}\left(2\cdot \frac{T_{\mathrm{melt}}-{\bar{T}}_{\mathrm{mold}}}{{\hat{T}}_{e}-{\hat{T}}_{\mathrm{mold}}}\right)\;$$

where D [m] represents the distance between the surface of the plastic part and the collimator core, geometric region of the plastic part with the highest temperature (see Figure 17), T_melt_ [°C] represents the injection temperature of the plastic front molten (see Table 8), ${\hat{T}}_{e}$ [°C] represents the maximum demoulding temperature in the collimator core of the plastic part (see Figure 17), and ${\bar{T}}_{\mathrm{mold}}$ [°C] represents the average temperature on the surface of the cavity of the mold (see Figure 17).

**Figure 18:**
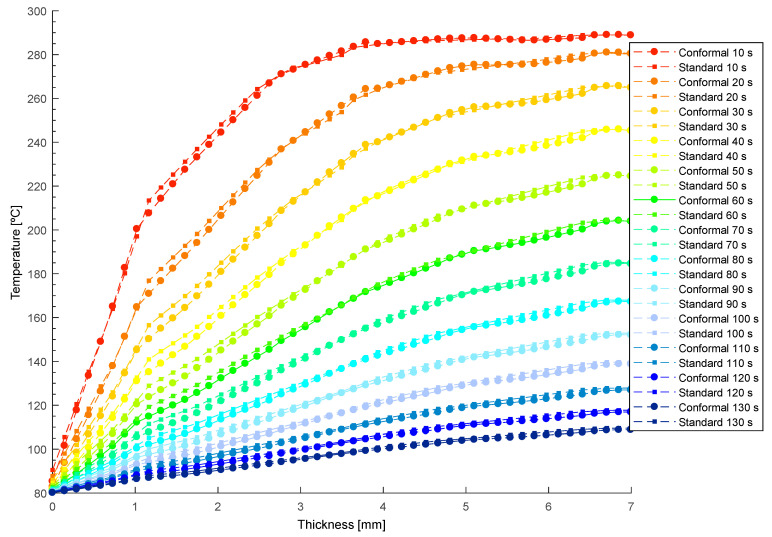
Temperature profile of the plastic part [°C] versus the thickness [mm] resulting in the collimator region during the transient numerical analysis.

According to Equation (6), it can be verified that the time that elapses until the plastic part reaches the ejection temperature depends closely on the temperature profile inside and on the surface of the plastic part. However, the temperature map of the plastic part is developed in a transient regime until reaching the regime or a stationary solution for the manufacturing process of the plastic part. This means that the distribution and magnitude of the temperature map of the plastic part varies as a function of the time variable, until a stationary solution is reached. Figure 18 shows the evolution for different instants of time in the numerical and transient analysis of the cooling phase of the plastic part of the temperature profile versus the thickness of the plastic part in the core area of the central collimator. This region is determined to be the most unfavorable, as it has a greater thickness, a higher temperature, and, therefore, it will be the region of the plastic part that requires the longest cooling time (see Equation (6)). In this way, the ejection phase of the plastic part will be conditioned to the instant in which the ejection condition is fulfilled for this region of the collimator (see Equation (7)).

(7) $${\bar{T}}_{e}\leq T_{\mathrm{ejection}}$$

where ${\bar{T}}_{e}$ [°C] represents the integral mean temperature of the temperature profile (see Figure 17) in the collimator region, and T_ejection_ [°C] represents the ejection temperature recommended by the manufacturer of the plastic material that was used for the present case study (see Table 8).

**Figure 19:**
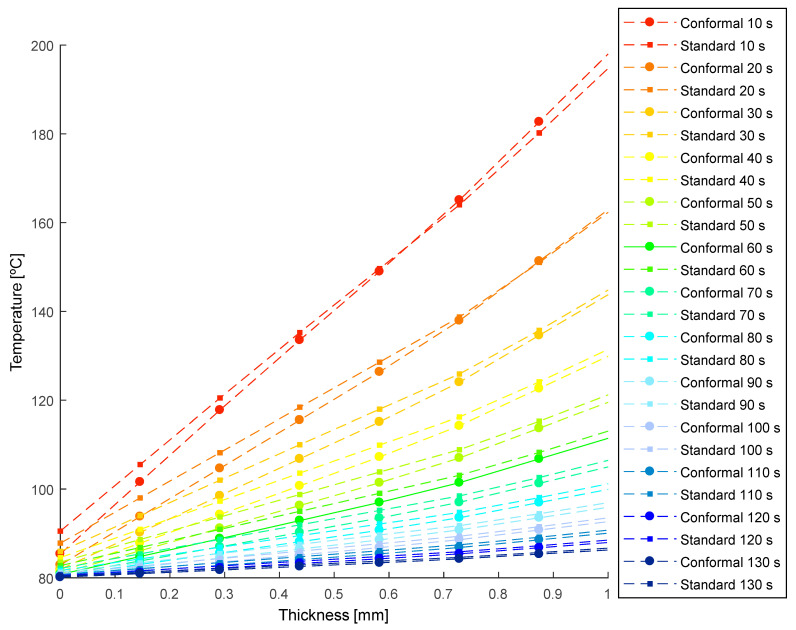
Temperature profile of the plastic part [°C] versus the thickness [mm] resulting in the collimator region during the transient numerical analysis.

Figure 18 and Figure 19 show the temperature profile obtained, in the collimator region, after transient thermal analyses for the defined cooling systems (see Figure 15 and Figure 16). In this way, from Figure 18 and Figure 19, the thermal performance of both systems can be compared, and the influence of the geometry and typology of the cooling channels used can be evaluated. As shown in Figure 18 and Figure 19, conformal-type cooling channels reduce the surface temperature of the plastic part, ${\bar{T}}_{\mathrm{mold}}$ [°C], in the collimator region. Moreover, this trend is maintained, significantly, up to thicknesses comprised in the range of 4 to 5 mm. From this range, the temperature profiles tend to be similar. However, the magnitude of the temperature of the plastic part in the collimator core is still lower for the design of the conformal-type cooling system.

Likewise, from the results shown in Figure 18 and Figure 19, it is also possible to define the magnitude of the temperatures ${\hat{T}}_{e}$ [°C] maximum demoulding temperature in the core of the plastic part, and ${\bar{T}}_{\mathrm{mold}}$ [°C], the mean temperature on the surface of the mold cavity (see Equation (6)).

**Figure 20:**
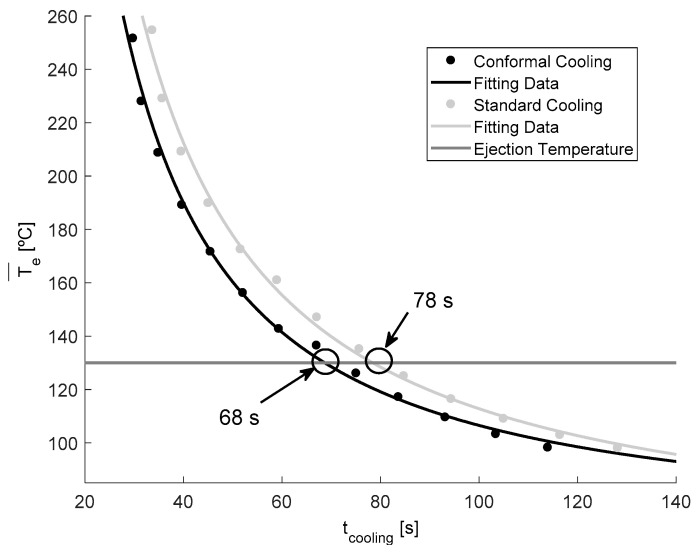
Cooling time [s] versus mean integral temperature [°C].

In this way, according to Equation (6), the associated cooling time can be calculated for each time point evaluated for both the designs of the cooling system. Finally, for each instant of time analyzed in the numerical simulations, the cooling time and the integral mean temperature associated with each temperature profile can be determined (see Figure 20).

As shown in Figure 20, from the technological condition defined to start the ejection phase (see Equation (7)), which is established when the mean integral temperature of the temperature profile is equal to, or lower than, the ejection temperature of the plastic material, the total time until the plastic part reaches the ejection temperature is calculated for both the designs of the cooling system. Table 9 shows and compares the magnitude of the technological parameters evaluated for the traditional cooling system design versus the conformal-type cooling system, which was proposed for the case study analyzed in this paper.

**Figure 21:**
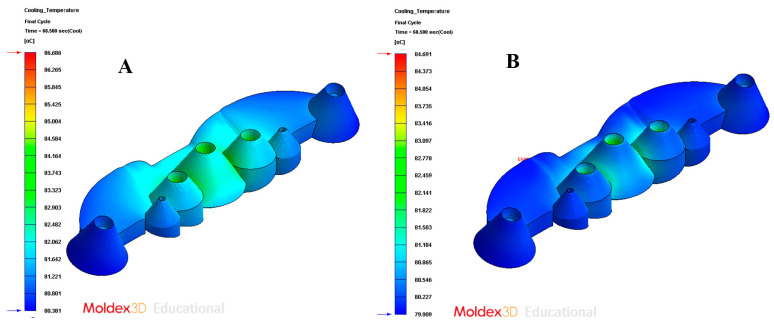
Cooling temperature. (**A**) Traditional cooling; (**B**) conformal cooling.

As Table 9 shows, for the design of the conformal-type cooling system, the cooling time obtained is equal to 68 s. While for the standard-type cooling design, the obtained cooling time is equal to 78 s. Therefore, the application of a conformal-type cooling system design reduces, for the case study analyzed in this paper, 10 s and 13% the manufacturing cycle time of the total plastic part. Likewise, taking into account the other technological parameters analyzed, it can be determined that the application of a conformal-type cooling design, for the case study analyzed in this paper, reduces the temperature on the surface of the mold cavity by 1.8 °C, the maximum temperature at the core of the collimator geometry of the plastic part is reduced by 0.7 °C, and the temperature gradient along the surface of the mold is reduced by 0.7 °C. It should be noted that, as shown by the results obtained after the numerical analyses performed (see Figure 21A,B), the conformal-type cooling system improves uniformity throughout the temperature map of the plastic part. This reduces, a posteriori, the gradients, concentrators, and stress distribution associated with temperature changes along the geometric domain of the plastic part.

**Figure 22:**
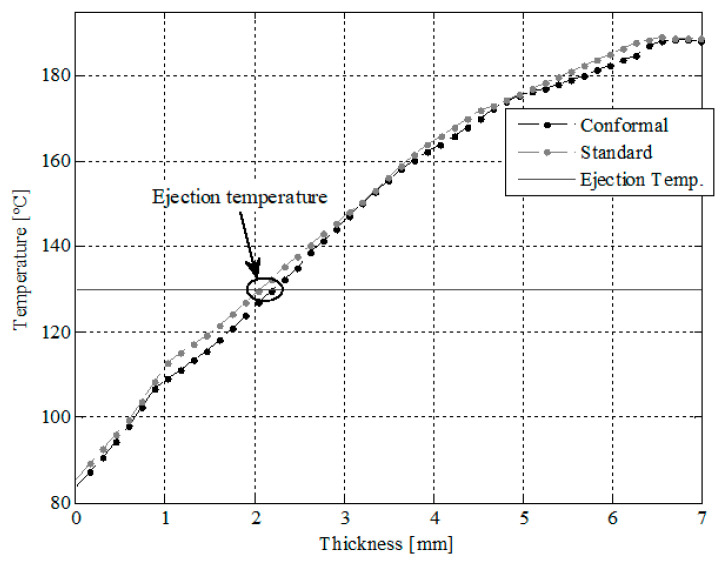
Temperature profile [°C] versus thickness [mm] in the collimator region for the beginning of the ejection phase of the plastic part.

**Figure 23:**
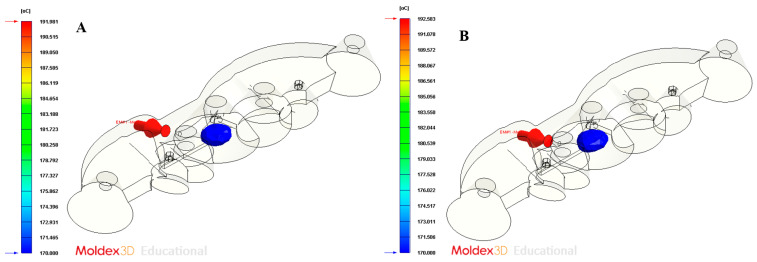
Molten core at the ejection time. (**A**) Traditional cooling; (**B**) conformal cooling.

Figure 22 shows the temperature profile of the plastic part versus the thickness for the geometric region of the collimator, at the instant in which the plastic part is ejected. As can be observed in Figure 21, the amount of solidified thickness can be evaluated and that, in addition, it is below the ejection temperature of the plastic material. In particular, for the design of the conformal-type cooling system, the ejection temperature of the plastic material is reached for a thickness in the geometric region of the collimator of 2.0 mm. Whereas, for the design of the traditional-type cooling system, the ejection temperature of the plastic material is reached for a thickness in the geometric region of the collimator of 2.2 mm. Therefore, the use of a conformal-type cooling system, for the case study analyzed in this manuscript, also reduces the amount of thickness in the core of the collimator, which is found to be above the ejection temperature of the plastic material (see Figure 23A,B, and Table 10).

### 3.3. Comparison of the Thermal Variables for the Proposed Cooling Systems

**Figure 24:**
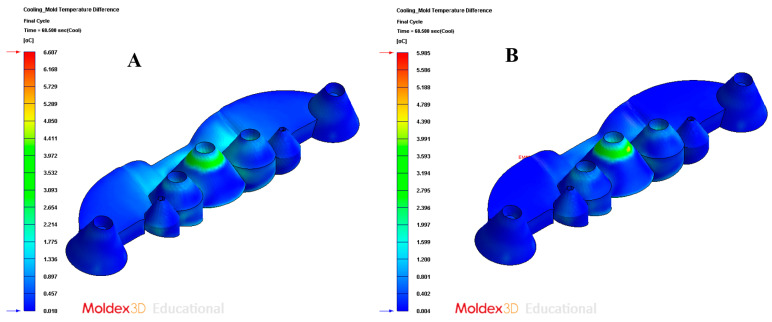
Mold temperature difference. (**A**) Traditional cooling; (**B**) conformal cooling.

**Figure 25:**
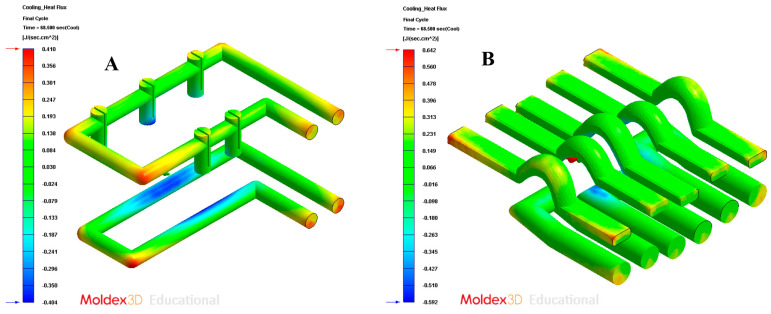
Cooling heat flux. (**A**) Traditional cooling; (**B**) conformal cooling.

**Figure 26:**
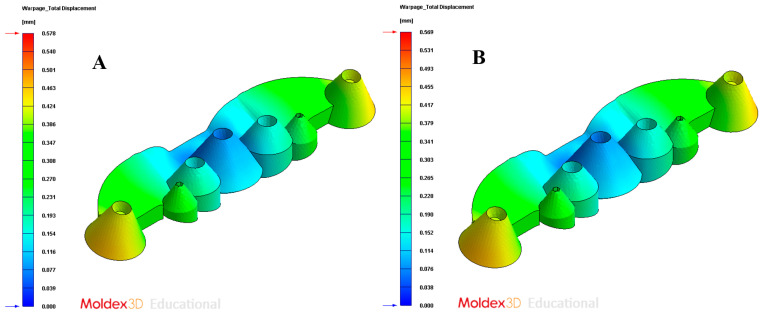
Total warpage displacements. (**A**) Traditional cooling; (**B**) conformal cooling.

**Figure 27:**
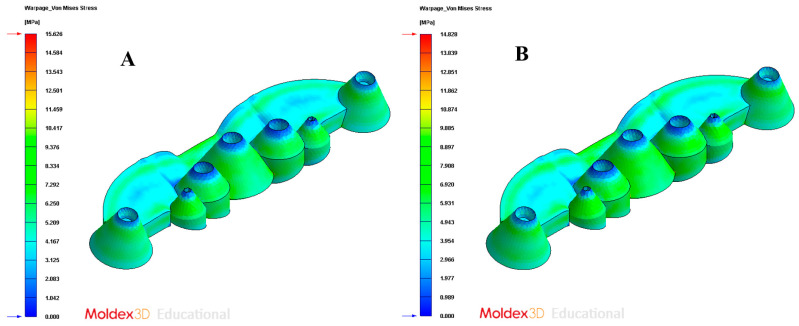
Warpage von Mises stress. (**A**) Traditional cooling; (**B**) conformal cooling.

In order to validate the improvement in the thermal performance presented by the proposed parametric design of the conformal-type cooling channels compared to the current conventional design of the cooling system, for the case study analyzed, the results obtained from the transient numerical analyses that were performed have been compared for the following variables: temperature map along the surface of the plastic part (see Figure 21A,B), temperature gradient along the surface of the plastic part (see Figure 24A,B), heat flux transferred from the plastic part to the cooling channels (see Figure 25A,B), displacements in the surface of the plastic part plastic associated with temperature gradients (see Figure 26A,B), and von Mises stresses associated with temperature gradients (see Figure 27A,B). Likewise, Table 11 shows the magnitude of the maximum values obtained for each variable analyzed, in each design of the cooling system proposed, together with the percentage of improvement that has been obtained by applying the design and solution of the conformal-type parametric cooling channels. It should be noted that the magnitude of said maximum values of each variable has been established for each design of the channels of the cooling system, from its corresponding previously defined cooling time. That is to say that the improvement in the thermal performance of the design of the parametric cooling channels of the conformal type not only has a relevant reduction in the cycle time or manufacturing of the plastic part, but, in addition, it also improves the uniformity of the temperature map on the surface of the plastic part, and the field of displacements and stresses associated with the temperature gradient on the surface of the plastic part, despite the difference in cooling time between the two solutions being 10 s.

## 4. Conclusions

The paper presents a new parameterized design of conformal cooling channels, for application in collimator-type plastic optical parts. The conformal channels that are presented exceed the thermal and dynamic performance of traditional and standard conformal channels, since they implement channels with new sections of complex topology that are capable of meeting the high geometric and functional restrictions of the optical part, as well as the technological requirements of the additive manufacturing of mold cavities. In order to evaluate the improvement and efficiency of the thermal performance of the presented solution, a transient numerical analysis of the cooling phase has been carried out, comparing traditional cooling with the new conformal geometry proposed. For this, the evolution of the temperature profile versus the thickness of the piece in the region of the collimating core with greater thickness and temperature, has been evaluated in a transient mode; this being the most unfavorable area from the point of view of cooling. The analysis of the thermal profiles, the calculation of the mean integral temperature of ejection at each point of the transient analysis, and the use of the Fourier formula, show the great improvement in the cycle time of the solution presented, compared to traditional cooling. The application of the conformal design that is presented in the paper reduces the production cycle time by 10 s, with this value being 13% of the total manufacturing cycle of the plastic part. Additionally, the use of the cooling system proposed in the paper reduces the amount of thickness in the core of the collimator, which is above the expulsion temperature of the plastic material.

The improvement in the thermal performance of the design of the parametric cooling channels presented not only has a significant reduction in the cycle time, but also improves the uniformity in the temperature map of the surface of the collimating piece, the field of displacements, and the stresses associated with the temperature gradient on the surface of the optical part.

The new cooling system manages to optimize, in a highly efficient way, the thermal exchange between the coolant flow and plastic material, fundamentally in the hot spots of the piece, and areas with a high thickness ratio and minimal accessibility. The new geometry that is presented in the paper improves the state-of-the-art, since it optimizes, on the one hand, the heat exchange between the fluid and the surface of the part in those hot spots of the collimating part that are impossible to cool with standard conformal cooling circuit layouts, and, on the other hand, greatly reduces the production costs of the manufacturing of collimating parts, which is currently one of the parts with the greatest expansion in the area of optics for automotive LED headlamps.

## Figures and Tables

**Table 1:** Parameters of the new conformal cooling design for collimators.

Parameter	Description	Units
D_p_	Distance from the cooling channel to the surface of the part	mm
D_ch_	Distance between the first cooling surface and the second cooling surface	mm
H_ch_	Height input conformal channels	mm
S_ch_	Conformal cooling inlet size	mm
W_ch_	Conformal cooling channels width	mm
a_ch_	Conformal cooling channel dome inclination	
L_ch_	Conformal channel length	mm

**Table 2:** Parameters of the cavity plate cooling system.

Parameter	Description	Units
C_ch_	Central channel vertical separation	mm
V_ch_	Lateral channels vertical separation	mm
F_ch_	Distance between inlet and outlet central channels	mm
G_ch_	Distance between inlet and outlet side channels	mm
P_ch_	Lower cooling section diameter	mm

**Table 3:** Dimensional values of the design parameters used in the cooling of the collimating part in the core of the mold.

Parameter	Units	Central Channel	Lateral Channels	End Channels
D_p_	mm	5	5	5
D_ch_	mm	5	5	5
H_ch_	mm	4	8	4
S_ch_	mm	5	5	5
W_ch_	mm	11	11	11
a_ch_	◦	60	60	60
L_ch_	mm	50	50	50

**Table 4:** Dimensional values of the design parameters used in the cooling of the collimating part in the mold cavity.

Parameter	Units	Central channels	Lateral Channels
C_ch_	mm	11	
V_ch_	mm		11
F_ch_	mm	17	
G_ch_	mm		16
P_ch_	mm	10	10

**Table 5:** Magnitude of the physical properties of the defined materials for the numerical simulations.

Nomenclature	Units	Description	Water (Pure)	Makrolon LED 2245 (PC)	Steel Alloy 1.2709
ρ_w_, ρ_p_, ρ_s_	kg/m^3^	Density	988	1051	8000
C_w_, C_p_, C_s_	J/kg·°C	Specific heat	4180	1682	450
δ_w_, δ_p_, δ_s_	W/m·°C	Thermal conductivity coefficient	0.643	0.253	20

**Table 6:** Thermoplastic material definition for the numerical simulations.

Description	Units	Makrolon LED 2245 (PC)
Material type	-	Polycarbonate
Supplier	-	Covestro
Fiber percent	%	0.0
Viscosity model	-	Modified Cross Model
PVT model	-	Modified Tait Model
Mechanical model	-	Isotropic pure polymer
Viscoelastic model	-	White–Metzner
Melt temperature	°C	320.0–280.0
Mold temperature	°C	120.0–80.0
Ejection temperature	°C	130
Freeze temperature	°C	170

**Table 7:** Mesh statistics for the traditional and conformal cooling meshes.

Description	Units	Traditional Cooling	Conformal Cooling
Part mesh node count	-	61,055	58,892
Part mesh element count	-	174,581	173,142
Part mesh volume	m^3^	1.068 × 10^−5^	1.068 × 10^−5^
Runner mesh node count	-	3133	3615
Runner mesh element count	-	2688	3136
Runner mesh volume	m^3^	1.000 × 10^−8^	1.000 × 10^−8^
Plastic part precisión (ε)–Mesh sizing	mm	1.000	1.000
Element type	-	Tetrahedral (10 nodes)	Tetrahedral (10 nodes)
Element type–Boundary layers	-	Prism (15 nodes)	Prism (15 nodes)
Offset ratio–Boundary layers	-	0.1	0.1

**Table 8:** Technological variables defined for the set-up of the filling and cooling stage for numerical simulations.

Nomenclature	Units	Description	Study Cases–Makrolon LED 2245 (PC)
t_fill_	s	Filling time	4.0
t_pack_	s	Packing time	8.5
t_cooling_	s	Cooling time	120.0
T_melt_	°C	Melt temperatue	290.0
T_mold_	°C	Mold temperature	80.0
T_eject_	°C	Ejection temperature	130.0
T_coolant_	°C	Coolant temperature	80.0
P_inj_	MPa	Maximum injection pressure	160.0
P_pack_ (t_pack_)	MPa	Packing pressure profile	85 (0.00 s–8.00 s) 40 (8.00 s–8.25 s) 10 (8.25 s–8.50 s)
P_coolant_	MPa	Coolant pressure	0.5

**Table 9:** Magnitude of the technological parameters evaluated for the traditional and conformal type refrigeration design, in the geometric region of the collimator.

Nomenclature	Units	Description	Traditional Cooling	Conformal Cooling
t_cooling_	s	Cooling time	78.0	68.0
${\bar{T}}_{\mathrm{mold}}$	°C	Mold wall temperature	82.4	80.6
${\hat{T}}_{e}$	°C	Maximum demolding temperature	189.1	188.4
ΔT_mold_	°C	Mold temperature difference	6.6	5.9

**Table 10:** Molten core dimensions and volume at the ejection time for the conformal and traditional cooling systems.

Nomenclature	Units	Description	Traditional Cooling	Conformal Cooling	Performance Improvement
d_molten x_	mm	Axis X molten core dimension	7.2	6.9	0.3 mm—4.5%
d_molten y_	mm	Axis Y molten core dimension	8.7	8.5	0.2 mm—2.4%
d_molten z_	mm	Axis Z molten core dimension	3.9	3.8	0.1 mm—2.6%
V_molten_	mm^3^	Molten core volume	127.9	109.5	18.4 mm^3^—16.8%

**Table 11:** Comparison of the maximum values of the thermal and mechanical variables obtained after the numerical analysis for each cooling system design.

Description	Units	Traditional Cooling	Conformal Cooling	Performance Improvement
Cooling temperature	°C	86.686	84.691	2.36%
Mold temperature difference	°C	6.607	5.985	10.39%
Cooling heat flux	J/s·cm^2^	0.410	0.642	56.58%
Total warpage displacement	mm	0.578	0.568	1.58%
Warpage von Mises stress	MPa	15.626	14.828	5.38%

## Data Availability

All data included in this study are available upon request by contact with the corresponding author.
